# Nonlinear relationship between early life stress exposure and subsequent resilience in monkeys

**DOI:** 10.1038/s41598-019-52810-5

**Published:** 2019-11-07

**Authors:** Karen J. Parker, Christine L. Buckmaster, Shellie A. Hyde, Alan F. Schatzberg, David M. Lyons

**Affiliations:** Department of Psychiatry and Behavioral Sciences, Stanford University School of Medicine, Stanford, CA 94305 USA

**Keywords:** Animal behaviour, Depression

## Abstract

Retrospective correlational studies of humans suggest that moderate but not minimal or substantial early life stress exposure promotes the development of stress inoculation-induced resilience. Here we test for a nonlinear relationship between early life stress and resilience by comparing varying “doses” of early life stress. Juvenile squirrel monkeys underwent one of five treatment conditions between 17–27 weeks of age: Stress inoculation (SI) with continuous access to mother (SI + Mom; one stress element), SI without continuous access to mother (SI; two stress elements), SI without continuous access to mother and with alprazolam injection pretreatments (SI + Alz; three stress elements), SI without continuous access to mother and with vehicle injection pretreatments (SI + Veh; three stress elements), or standard housing (No SI; zero stress elements). Alprazolam was used to test whether anxiolytic medication diminished SI effects. Subjects exposed to one or two early life stressors subsequently responded with fewer indications of anxiety (e.g., decreased maternal clinging, increased object exploration, smaller cortisol increases) compared to No SI subjects. Subjects exposed to three early life stressors did not differ on most measures from one another or from No SI subjects. These findings provide empirical support for a nonlinear J-shaped relationship between early life stress exposure and subsequent resilience.

## Introduction

Early life stress exposure is commonly viewed as increasing the risk of later manifestations of psychopathology^1–3^. Interestingly, however, early life stress exposure also appears to promote the development of subsequent resilience. For example, moderate adversity in childhood has been linked to lower subsequent levels of state anxiety^4^ and a smaller increase in the salivary cortisol response to the Trier Social Stress Test^5^. Prior moderately stressful experiences diminish emotional distress in workplace conditions^6^ and decrease cardiovascular responses to stressful laboratory tests^7^. Moderate amounts of lifetime adversity have also been associated with lower global distress, fewer trauma symptoms, and higher life satisfaction in adults^8,9^. These findings suggest that exposure to moderate but not minimal or substantial early life stress has an inoculation-like effect that enhances subsequent emotion regulation and resilience as has been depicted by a non-linear J-shaped function^10,11^.

Little is known about the mechanisms that mediate stress inoculation-induced emotion regulation and resilience. Programs for promoting resilience in children tend to focus on parent-child relationships^12^ or rigorous preschool training interventions^13^ that seldom directly consider the possibility that cognitive and emotional engagement and mastery of moderate adversity are necessary conditions for intervention-based changes in behavior to occur. We and other investigators have argued that resilience arises from intermittent exposure to early life stressors that are not overwhelming, but challenging enough to evoke acute anxiety and transiently activate the hypothalamic-pituitary-adrenal (HPA) axis^12,14–17^. States of moderate arousal enhance learning^18^, and may thereby render emotion regulation during subsequent coping efforts more proficient. In rodents, anxiolytic medications interfere with learned extinction of conditioned fear^19^, whereas the anxiogenic compound yohimbine activates the HPA axis and accelerates learned extinction of conditioned fear^20^. These findings suggest that anxiolytic pretreatment prior to moderate early life stress exposure may disrupt the development of resilience.

The present research extends our understanding of the effects of early life stress exposure in two respects. First, we experimentally test for a nonlinear relationship between early life stress exposure and subsequent indications of resilience in a non-human primate model. Varying “doses” of early life stress exposure were studied in a prospective, controlled design as detailed below. We also consider whether pretreatment with the anxiolytic benzodiazepine alprazolam prior to repeated early life stress exposure disrupts the development of emotion regulation measured using procedures established in our earlier research. Alprazolam produces fast-acting effects that do not appear to diminish with repeated administration^21^, and has a broader range of anxiolytic effects compared to other available benzodiazepine medications in monkeys^22^.

## Methods

### Subjects

A total of seventy-four squirrel monkeys (*Samiri sciureus*) that were born at the Stanford University Research Animal Facility served as subjects (46 females, 28 males). All subjects were maintained in undisturbed natal groups through 16 weeks of age. Group composition was determined by birth dates to minimize developmental differences between the monkeys in each natal group. Seasonal synchronous breeding facilitated the generation of age-matched groups.

Groups were housed indoors in 1.8 × 1.2 × 1.8-m species appropriate cages that were cleaned daily. Housing and testing occurred in climate-controlled rooms with an ambient temperature of 26 °C. Light/dark cycles were 12:12 hours with lights on at 0700 hours. All monkeys were provided unrestricted access to fresh drinking water and monkey chow (LabDiet 5040, PMI Nutrition International, St. Louis, Missouri, USA) with fruit and vegetable supplements. Various toys, swinging perches, and simulated foraging activities were provided for environmental enrichment. To facilitate husbandry-related activities and the experimental manipulations, monkeys were trained to leave the home cage through a small sliding door connected to a transport box used for capture and transportation. All procedures were approved by Stanford University’s Administrative Panel on Laboratory Animal Care and were carried out in accordance with the National Institutes of Health Guide for the Care and Use of Laboratory Animals.

### Experimental design

**Figure 1:**
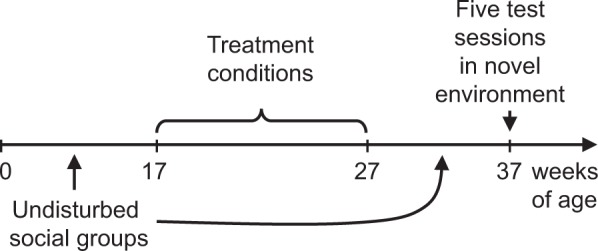
Schematic overview of the experimental design and study timeline. Subjects (N = 62) underwent one of five treatment conditions, with varying stress elements, between 17 and 27 weeks of age. Subjects were assessed 10 weeks later, at 37 weeks of age, for behavioral and neuroendocrine indications of resilience.

A schematic overview of the study timeline is provided in Fig. 1. Experimental subjects were run in two different cohorts (N = 62) as described below. Subjects underwent one of five 10-week treatment conditions that occurred between 17 and 27 weeks of age: Stress inoculation (SI) with continuous access to mother (SI + Mom, N = 10; one stress element), SI without continuous access to mother (SI, N = 11; two stress elements), SI without continuous access to mother and with alprazolam injection pretreatments (SI + Alz, N = 10; three stress elements), SI without continuous access to mother and with vehicle injection pretreatments (SI + Veh, N = 11; three stress elements), or standard laboratory housing (No SI, N = 20; zero stress elements). Subjects in the No SI condition were maintained in undisturbed groups. Subjects in the other four treatment conditions were temporarily separated from natal groups once a week for 10 total 1-hour sessions that entailed exposure to different stress elements as summarized in Table 1. After each session, subjects were returned to the home cage and reunited with the natal group. All sessions occurred between 1215 and 1745 hours, and no more than one monkey from each natal group was separated on a given day. After completion of the treatment conditions at 27 weeks of age, all subjects were maintained in undisturbed groups under identical laboratory conditions. Puberty occurs between 2–3 years of age and the average maximum lifespan in captivity is approximately 21 years^23^.

**Table 1:** Treatment conditions differed in terms of three elements of stress inoculation.

Treatment condition	Separated from natal group	Separated from mother	Injection pretreatment	Number of early life stressors
No SI	No	No	No	0
SI + Mom	Yes	No	No	1
SI	Yes	Yes	No	2
SI + Veh	Yes	Yes	Yes	3
SI + Alz	Yes	Yes	Yes	3

Before each session in the SI + Alz treatment condition, subjects were pretreated with an intramuscular injection of 100 µg/kg alprazolam in 40% propylene glycol, 10% ethyl alcohol, 0.9% benzyl alcohol, and sterile water prepared by the Drug Product Services Laboratory at the University of California, San Francisco, Medical Center Pharmacy. Subjects were then returned to the home cage for 15 minutes and thereafter removed from the mother and natal group for a 1-hour separation session. Dose response studies were conducted in a separate (third) cohort of N = 12 monkeys to determine the alprazolam dose needed to attenuate behavioral and neuroendocrine indications of stress during social separations with minimal ataxic side effects as described in the Supplemental Information. To control for injection pretreatment effects, subjects were administered vehicle instead of alprazolam before each session in the SI + Veh treatment condition.

Subjects in both the SI and SI + Mom conditions were not pretreated with alprazolam or vehicle injections and were included from previous studies^15,24^, to determine whether injection pretreatments modulate separation effects. These two conditions were identical except that in the SI + Mom condition, each subject and its mother were removed together from natal groups for each session. Subjects from the other treatment conditions were separated from both the mother and natal group as summarized in Table 1. Ten of the 20 No SI subjects were included from our earlier SI studies^15,24^ as cohort-matched controls. Subjects in the No SI condition from earlier studies did not differ significantly from those raised for the present study and therefore these cohorts were combined. Thus, the present study’s animals were produced in two separate cohorts (i.e., cohort 1: SI + Mom, SI, and No SI subjects; cohort 2: SI + Alz, SI + Veh, and No SI subjects). It is important to note that we decided not to produce additional SI and SI + Mom subjects for the present study for the purposes of replicating an effect that we and others have shown in multiple independent studies^24–26^. This decision was guided by the ethical principles of the “3 R’s” (Replace, Reduce, Refine)^27^, and served to significantly reduce the number of monkeys generated for the present study while still allowing us to employ the same rigorous methodology and test our overall stress “dosing” hypothesis.

### Follow-up tests and measures

Ten weeks after completion of the treatment conditions, each subject was tested along with its mother in a novel environment to assess anxiety-like behavior and plasma levels of cortisol. The novel test cage (60 × 60 × 90 cm) and unfamiliar room used for testing differed from those used for all four SI conditions. The cage was cleaned between tests and contained food, drinking water, perches, and various toy-like objects. Each subject was tested in drug-free conditions for 30 minutes each day at the same time of day for a total of five repeated test sessions.

During each test session, a trained observer seated in plain view of the monkeys used a computer-assisted data entry device (Observer XT, Noldus Information Technology; Leesburg, VA) to score four aspects of behavior: (1) total duration of maternal clinging, i.e., time spent in the species-typical riding posture on the mother’s shoulders and upper back, (2) latency to first terminate maternal contact, (3) latency to first explore a toy-like object scored when a subject touched an object, and (4) total object exploration counts.

Immediately after the first and last 30-minute test sessions, blood samples were collected to determine cortisol levels using previously described procedures^24^. Additional samples were collected 12 days before the first test session to provide baseline assessments in undisturbed home cage conditions. Most samples (84%) were collected within 180 seconds from cage entry (median = 124 seconds, range 60–342 seconds), and all but 7 samples (4%) were collected within 240 seconds. Sample collection latencies within this range account for less than 1% of the variance in squirrel monkey cortisol levels^28^.

After collection, each sample was centrifuged at 4 °C for 10 minutes and the plasma fraction was stored at −80 °C. Cortisol was measured in duplicate using a previously described radioimmunoassay^28^. Complete sample subsets from each treatment condition were included in every assay. Assays were performed by trained personnel without knowledge of the treatment or test conditions. Intra- and inter-assay coefficients of variation were, respectively, 6% and 7%, and assay sensitivity was 3 µg/dl.

### Data analysis

Composite measures of behavior were generated from z-scores based on Pearson correlation coefficients. Treatment effects were then assessed using least squares estimates for linear and quadratic functions in the MGLH module of Systat (Chicago, IL). Treatment condition was a between subjects factor and test session was a repeated within-subjects factor. The study was not adequately powered to detect sex-by-treatment interactions but sex main effects were examined as a statistical covariate. Post hoc pairwise comparisons with the No SI control condition were assessed with Fisher LSD tests. All test statistics were evaluated with two-tail probabilities (P < 0.05). Associations between composite measures were assessed with Pearson correlation coefficients.

## Results

**Figure 2:**
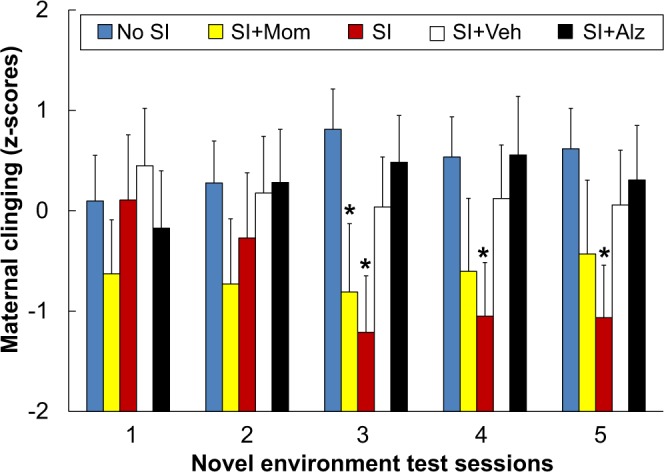
Treatment condition differences in maternal clinging. Maternal clinging scores measured in drug-free conditions during repeated novel environment test sessions are presented as mean ± SEM. Treatment conditions within each test session are ordered in terms of increasing exposure to more stress elements as described in Table 1. Asterisks indicate a significant difference relative to the No SI condition (P < 0.05).

Individual differences in the duration of maternal clinging and latency to first terminate maternal contact were highly correlated in each of the five test sessions (mean r = 0.84, df 60, P < 0.001). Standardized z-scores for these measures were added together to generate a maternal clinging score for each subject in each test session. Subjects from all treatment conditions were initially similar but differences emerged over repeated test sessions as discerned by a treatment-by-session interaction (F(16,224) = 1.83, P = 0.029). As depicted in Fig. 2, subjects from the SI and SI + Mom conditions responded with fewer indications of anxiety inferred from decreased maternal clinging compared to control subjects from the No SI condition during the last three test sessions. Subjects in the SI + Alz and SI + Veh conditions did not differ significantly from one another or from subjects in the No SI condition. Significant sex differences were not discerned in maternal clinging scores.

**Figure 3:**
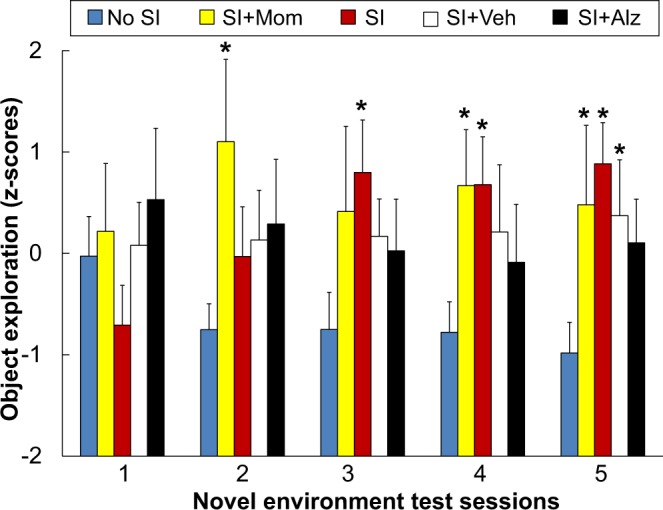
Treatment condition differences in exploration. Object exploration scores measured in drug-free conditions during repeated novel environment test sessions are presented as mean ± SEM. Treatment conditions within each test session are ordered in terms of increasing exposure to more stress elements as described in Table 1. Asterisks indicate a significant difference relative to the No SI condition (P < 0.05).

Individual differences in the latency to first explore an object and object exploration counts were inversely correlated (mean r = −0.58, df 60, P < 0.001). Standardized z-scores for these measures were used to generate an object exploration score for each subject in each test session. Subjects from all treatment conditions were initially similar but differences emerged over repeated test sessions as discerned by a treatment-by-session interaction (F(16,224) = 1.78, P = 0.036). As depicted in Fig. 3, subjects from the SI and SI + Mom conditions responded with increased object exploration compared to No SI subjects during the last four test sessions. Subjects from the SI + Veh condition also responded with increased exploration compared to No SI subjects during the last test session. Subjects from the SI + Alz condition did not differ significantly from No SI subjects, and sex differences in exploration were not discerned. As expected, individual differences in object exploration scores were inversely correlated with maternal clinging scores (r = −0.71, df 60, P < 0.001).

**Figure 4:**
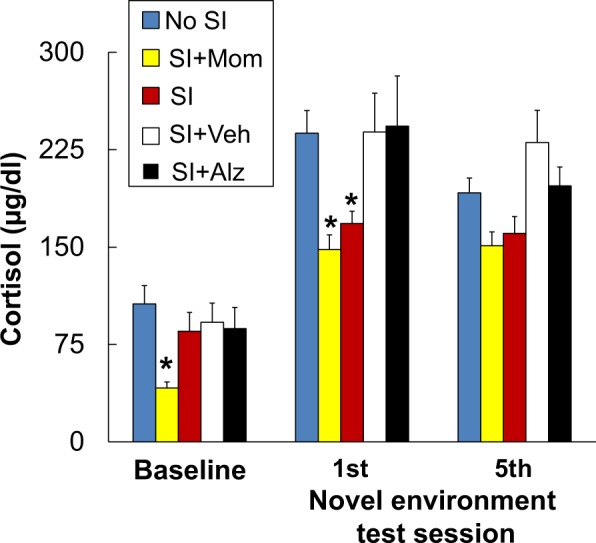
Treatment condition differences in cortisol. Plasma levels of cortisol measured in drug-free conditions before (baseline) and after the first and last novel environment test sessions are presented as mean ± SEM. Treatment conditions are ordered in terms of increasing exposure to more stress elements as described in Table 1. Asterisks indicate a significant difference relative to the No SI condition (P < 0.05).

Cortisol levels were consistently higher after the first (F(1,56) = 150.10, P < 0.001) and last test sessions (F(1,56) = 140.13, P < 0.001) compared to baseline levels measured in undisturbed home cage conditions for all groups. Treatment effects were also observed with smaller increases in cortisol after the first (F(4,56) = 3.46, P = 0.014) but not the last test session in monkeys from the SI and SI + Mom conditions compared to the No SI condition (Fig. 4). Baseline cortisol levels measured in undisturbed home cage conditions were likewise lower in subjects from the SI + Mom condition compared to the other conditions (F(4,56) = 2.89, P = 0.03). Subjects from the SI + Alz and SI + Veh conditions did not differ significantly from one another or from subjects in the No SI condition. Sex differences in cortisol levels were not discerned.

**Figure 5:**
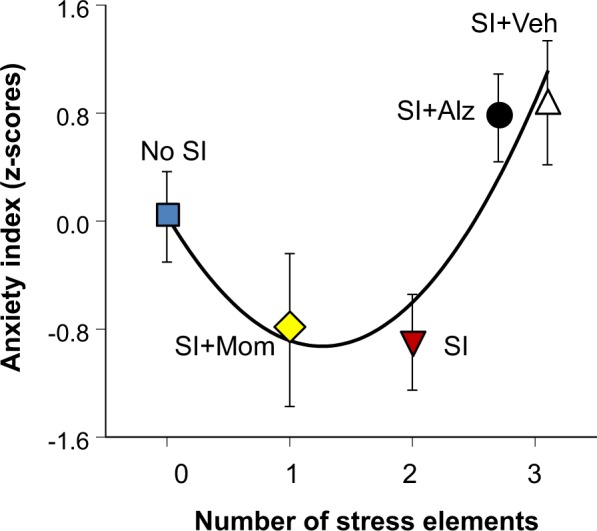
Treatment condition differences in evoked anxiety. Summary measure of anxiety evoked during the novel environment test sessions is plotted against the number of different stress elements in each treatment condition (mean ± SEM). Treatment conditions are ordered in terms of increasing exposure to more stress elements as described in Table 1. The quadratic J-shaped relationship was confirmed by statistical trend analysis (P = 0.001).

To more broadly determine whether the number of different stress elements in each treatment condition (Table 1) was related to subsequent responses observed over the five test sessions, each subject’s mean standardized post-test cortisol level, mean standardized maternal clinging score, and mean standardized object exploration score were used to generate a single summary index of evoked anxiety. Higher scores on this index reflect higher post-test cortisol levels, more maternal clinging, and diminished object exploration. Statistical trend analysis revealed that anxiety evoked during test sessions was not linearly related to the number of different stress elements in each treatment condition but instead followed a quadratic J-shaped function as depicted in Fig. 5 (F(2,59) = 7.19, P = 0.002; linear trend t = 1.70, P = 0.094; quadratic trend t = 3.38, P = 0.001).

## Discussion

Findings from this study indicate that monkeys exposed to one or two stressors in the SI + Mom and SI treatment conditions demonstrate diminished maternal clinging, enhanced object exploration, and smaller increases in cortisol during novel environment test sessions compared to monkeys from treatment conditions with fewer (No SI) or greater (SI + Alz and SI + Veh) numbers of early life stressors. Follow up analyses using a single summary index of evoked anxiety computed from standardized behavioral scores and post-test plasma cortisol levels confirmed that anxiety was nonlinearly related to the number of prior early life stressors. Findings from this prospective study of early life stress exposure in monkeys lend compelling experimental support for the nonlinear relationship observed between early life stress exposure and subsequent resilience in retrospective, correlational studies of humans^4,8^.

Young monkeys from all treatment conditions were initially similar in their behavioral response to the novel environment; they all exhibited high levels of maternal clinging and low levels of object exploration over the first several days of testing. Monkeys at this age rarely cling to their mothers^29^, and typically only do so to reduce arousal in the face of highly stimulating events^30^. Data from the first few days of testing suggest that the novel environment test served as a potent emotional challenge for offspring from all five rearing conditions.

Treatment-related differences in behavior gradually emerged over repeated test sessions such that subjects exposed to one or two stressors in the SI + Mom and SI treatment conditions exhibited less maternal clinging and more rapidly initiated object exploration during the latter days of testing compared to subjects previously exposed to fewer (No SI condition) or greater (SI + Alz and SI + Veh) numbers of stressors. This finding is consistent with the notion that prior experience with manageable stressors enhances emotion regulation under subsequently challenging circumstances^12,14–17^.

The neuroendocrine data from this study showed a similar effect, but a slightly different pattern, compared to the behavioral data. All monkeys exhibited significantly increased post-test cortisol levels above baseline conditions. However, subjects from the SI + Mom and the SI treatment conditions responded with smaller increases in cortisol levels after the first test session compared to subjects from the other treatment conditions with both fewer or greater numbers of stressors. Although cortisol levels in all monkeys remained elevated above baseline after the last test session, all groups showed a decline in stress induced-cortisol levels consistent with acclimation to the novel test environment, and no longer statistically differed from one another. Why treatment-related differences in behavior and HPA axis activation emerged over different time scales is unknown. This finding does suggest, however, that treatment-related differences in behavior are not directly attributable to concurrent differences in cortisol concentrations.

We previously hypothesized that evoked anxiety during SI may enhance learning and render emotion regulation during subsequent coping efforts easier and more proficient^15,16^. To test this possibility, alprazolam pretreatments were used to experimentally diminish anxiety evoked by SI as confirmed by dose response studies presented in the Supplemental Information. Two months after completion of the drug treatment protocols, subjects were tested in the emotionally challenging novel environment. As predicted, subjects from the SI + Alz treatment condition were behaviorally indistinguishable from No SI subjects. However, only on the last test session did subjects from the SI + Veh treatment condition exhibit increased exploration compared to subjects from the SI + Alz and No SI conditions. Subjects from the SI + Veh and SI + Alz treatment conditions were otherwise indistinguishable in maternal clinging and cortisol levels. These results provide only very modest support for the disruptive effects of alprazolam on the development of behavioral indications of resilience.

The data obtained from the SI + Veh condition are consistent with the nonlinear hypothesis of stress inoculation-induced resilience. Monkeys in this treatment condition were exposed to three different stressors, and it is possible that this substantial level of stress exposure obscured potential alprazolam treatment effects. Investigation of treatments with fewer stressors (e.g., SI + Mom + Veh versus SI + Mom + Alz) might therefore reveal more pronounced alprazolam disruption of SI effects.

The present study has several limitations that warrant discussion. First, we evaluated a fairly narrow range of possible treatment effects. Consequently, we do not know whether varying levels of early life stress exposure as studied here alter other aspects of the SI monkey phenotype, including performance on cognitive tests and growth of prefrontal cortical volumes across development^16,31^. However, there is evidence to suggest that variation in early life stress exposure has enduring effects, in at least some of these treatment groups: A neural correlate of enhanced cognitive control, dopamine D2 receptor availability in ventral striatum, is greater in subjects from SI + Mom and SI treatment conditions compared to No SI subjects assessed in mid-life adulthood at 11 years of age^32^. Enhanced cognitive control is an aspect of stress coping and emotion regulation in humans^33^ and impairments in cognitive control represent a dimension shared by diverse mental health disorders^34^. Second, it is likewise possible that alprazolam as administered here indeed disrupts other phenotypic outcomes, but these measures were not assessed in the present study, and we were unfortunately unable to study this cohort across lifespan development. Finally, the data used to identify stressful injection treatment effects in SI + Veh and SI + Alz monkeys were not obtained concurrently with the cohort of SI monkeys not injected with alprazolam or vehicle. The rationale for this decision was guided by ethical adherence to the principles of the 3 R’s as discussed above^27^. All procedures were conducted by the same team members and were identical for both cohorts, and the No SI monkeys included in each cohort did not differ on any measure. Nevertheless, stratification of treatment conditions to different cohorts represents a limitation of our study.

In conclusion, we tested for a causal nonlinear relationship between early life stress exposure and subsequent resilience. We also examined whether alprazolam disrupts SI-induced resilience. Clinical trials and preclinical studies of rodents suggest that alprazolam and other benzodiazepines may diminish the efficacy of psychotherapies^19,35,36^ but systematic reviews of the literature have not revealed clear-cut evidence^37–39^. We did not find support for alprazolam disruption of later resilience and show that resilience was not linearly related to the number of different stressors administered in each early life treatment condition but instead followed a quadratic J-shaped function. These findings dispel the notion that early life stress exposure is invariantly pathogenic, and provide experimental support for the view that moderate early life stress exposure enhances subsequent resilience. Research aimed at understanding the mechanisms that mediate these nonlinear effects of early life stress may provide new targets for interventions that enhance resilience and recovery from stress-related disorders in humans.

## Supplementary information

Supplementary Information

## Data Availability

Data are available upon request.
