# Supplementary material for: Nonlinear relationship between early life stress exposure and subsequent resilience in monkeys
Source: Sci Rep. 2019 Nov 7;9:16232. doi: 10.1038/s41598-019-52810-5 (PMC6838120; doi:10.1038/s41598-019-52810-5)
Supplement: Supplementary file 1 — Supplementary Information [file 41598_2019_52810_MOESM1_ESM.pdf]

SUPPLEMENTARY INFORMATION

Title: Nonlinear relationship between early life stress exposure and subsequent resilience in monkeys

Authors and Academic Affiliation: Karen J. Parker, Christine L. Buckmaster, Shellie A. Hyde, Alan F. Schatzberg, and David M. Lyons; Department of Psychiatry and Behavioral Sciences, Stanford University School of Medicine, Stanford, CA 94305 USA

Alprazolam Dose Response Studies

These studies were designed to determine the dose of alprazolam that most effectively attenuates stress-induced anxiety and hypothalamic-pituitary-adrenal (HPA) axis activation with minimal concomitant ataxic effects. Two studies were performed with 12 experimentally naïve juvenile squirrel monkey (N=6 males, 6 females) subjects. In each of these studies, a single dose of alprazolam was intramuscularly (IM) administered into the monkey's quadricep muscle 15 minutes before a 1 hour social separation period from the mother and natal group that was similar to the 1 hour sessions of stress inoculation (SI) described in the main text. It is well established that such social separations induce distress calls, locomotor agitation, and acute increases in cortisol concentrations in squirrel monkeys[1-3].

Monkeys were randomized to different dose orders according to a Latin square design. Tests for a given subject were spaced at least 1 week a part to provide an adequate drug "wash out" period. The wash out period was calculated based on knowledge of the half-life for alprazolam (11 hours; range: 6 to 26 hours) and evidence that 90% of drug clearance is achieved in 3.3 times the drug's half-life[4]. Testing occurred between 1330 and 1800 hours with a given monkey tested at the same time of day for each drug dose received. Up to three monkeys were tested each day (no two from the same natal group), and both studies were conducted by trained observers without knowledge of the treatment conditions.

Study 1 Methods. Each monkey (N=12) received four different doses of alprazolam (0, 50, 100, or 200 µg/kg) compounded for intramuscular injection in a vehicle solution as described in the main text. Two experimenters recorded behavioral indices of anxiety (i.e., on-the-signal locomotor activity; all occurrences of species-typical distress calls) and ataxia (i.e., all occurrences of perch stumbles) during the 1 h test session. The monkey was then rapidly removed from the test cage, manually restrained, and 0.8 ml of whole blood was collected via femoral venipuncture. Prior to the beginning of this study, blood samples were also collected

from subjects under undisturbed home cage conditions to establish baseline measures of cortisol. Blood was collected and processed, and cortisol assays were conducted as described in the main text. The effect of drug dose on behavior and cortisol levels was assessed with analysis of variance (ANOVA) and paired t-tests as appropriate using Systat Software (Chicago, IL). For all analyses, test statistics were evaluated with two-tail probabilities ($P < 0.05$).

Study 1 Results. The highest dose of alprazolam (200 μg/kg body weight) reduced anxiety but resulted in considerable ataxia and partial sedation, and was excluded from further consideration. Ataxia measures did not differ significantly across the lower two alprazolam doses (i.e., 50 and 100 μg/kg) and the vehicle control [$F(2,20)=2.41$, $P=0.147$]. The lowest dose did not differ significantly from the vehicle control in terms of reducing behavioral measures of anxiety or HPA axis activation (Figure S1). In the vehicle control condition, cortisol levels immediately after the 1-hr test session were 1.7-fold greater than baseline levels measured in undisturbed home cage conditions [paired-$t(11)=8.29$, $P<0.001$]. Pretreatment with 100 μg/kg alprazolam significantly diminished both behavioral measures of anxiety [distress calls: omnibus $F(2,20)=4.3$, $P=0.04$; pairwise comparison of 100 μg/kg vs. vehicle dose $P=0.017$; agitated locomotor activity: omnibus $F(2,20)=5.567$; $P=0.017$; pairwise comparison of 100 μg/kg vs. vehicle dose $P=0.002$; Figure S1). No alprazolam dose attenuated the adrenocortical stress response (data not shown).

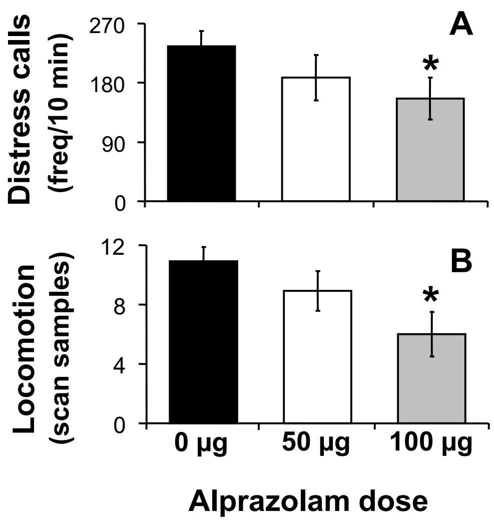

Figure S1. Alprazolam administration attenuates behavioral responses to a 1 hour social separation test. Dose response data are presented for (A) frequency of distress calls and (B) agitated locomotor activity scores (mean ± SEM). Asterisks depict significant differences ($P < 0.05$) between alprazolam (100 µg/kg) and vehicle control (0 µg/kg) treatment conditions.

Study 2 Methods. We extended the blood collection time course in Study 2 to test whether 100 µg/kg alprazolam compared to vehicle treatment affected post-stress measures of adrenocortical recovery after completion of the 1 hour test session. No behavioral measures were assessed in Study 2. Six juvenile monkeys (N=3 males, 3 females) served as subjects. Subjects closest to the mean for cortisol levels in Study 1 were selected for inclusion in Study 2. A one month period elapsed between the end of Study 1 and the beginning of Study 2. The injection protocol was identical to Study 1. Baseline blood samples were again collected prior to study initiation. Following blood collection immediately after the 1 hour test session (time point 0), each subject was returned to the home cage. Thereafter, each subject was rapidly recaptured and blood was collected 1 and 3 hours after time point 0. The blood volume for each

sample was 0.5 ml in Study 2, but otherwise, blood collection, cortisol assay procedures, and statistical analyses were the same as described above in Study 1 or in the main text.

Study 2 Results. Significantly lower levels of cortisol were maintained for at least 3 hours after the 1 hour test session in monkeys pretreated with 100 µg/kg alprazolam compared to the vehicle control [omnibus $F(2,8)=9.069$; $p=0.009$; Figure S2]. As in Study 1, alprazolam treatment did not alter the adrenocortical stress response at time point 0, but it did enhance adrenocortical recovery 1 hour (paired-$t(5)=4.09$, $P=0.009$) and 3 hours (paired-$t(5)=5.203$, $P=0.003$) after completion of social separation stress test compared to the vehicle control.

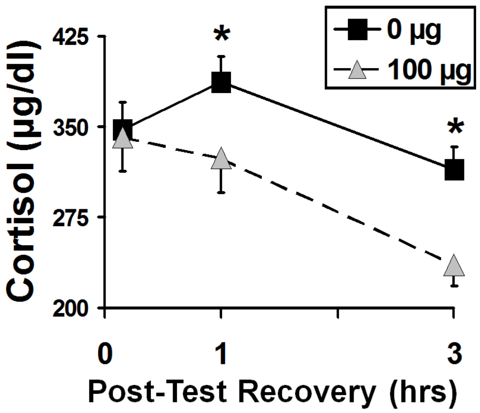

Figure S2. Alprazolam administration enhances recovery of plasma cortisol levels (mean ± SEM) after completion of the 1 hour social separation stress test. This finding is in keeping with the notion that alprazolam does not alter cortisol metabolism or clearance, but instead reflects anxiolytic effects. Asterisks depict significant differences ($P < 0.05$) between alprazolam (100 µg/kg) and vehicle control (0 µg/kg) treatment conditions.

Conclusions. These combined results indicate that a dose of 100 µg/kg alprazolam significantly attenuates acute anxiety and HPA axis recovery during social separation, but does not

completely abolish the behavioral or hormonal response. A complete blockade of anxiety achieved with a higher dose of alprazolam (200 µg/kg) produced the unwanted side effects of ataxia and partial sedation, which were not evident at lower doses (i.e., 50 or 100 µg/kg). Based on these findings, and similar dose-response data from humans[5], rhesus monkeys[6], and squirrel monkeys studied independently by other investigators[7], the 100 µg/kg dose of alprazolam was selected for use in the experiment described in the main text.

REFERENCES

1 Coe, C. L., Glass, J. C., Wiener, S. G. & Levine, S. Behavioral, but not physiological, adaptation to repeated separation in mother and infant primates. *Psychoneuroendocrinology* **8**, 401-409 (1983).

2 Hennessy, M. B. Multiple, brief maternal separations in the squirrel monkey: Changes in hormonal and behavioral responsiveness. *Physiology & Behavior* **36**, 245-250 (1986).

3 Stanton, M. & Levine, S. Brief separation elevates cortisol in mother and infant squirrel monkeys. *Physiology & Behavior* **34**, 1007-1008 (1985).

4 Facts and Comparisons. (Wolters Kluwer Health Inc, St. Louis, MO., June 2011).

5 Raj, A. & Sheehan, D. in *Textbook of Psychopharmacology* (eds A. F. Schatzberg & C. B. Nemeroff) 371-390 (American Psychiatric Publishing, Inc., Washington, D.C., 2004).

6 Kalin, N. H., Shelton, S. E. & Turner, J. G. Effects of alprazolam on fear-related behavioral, hormonal, and catecholamine responses in infant rhesus monkeys. *Life Sci* **49**, 2031-2044 (1991).

7 Birkett, M. A., Platt, D. M., Tiefenbacher, S., Bano, K. M. & Rowlett, J. K. Attenuation of the effects of a pharmacological stressor by alprazolam in squirrel monkeys. *Society for Neuroscience Abstract 7788.* (2005).
